# A study on the functions of ubiquitin metabolic system related gene FBG2 in gastric cancer cell line

**DOI:** 10.1186/1756-9966-28-78

**Published:** 2009-06-10

**Authors:** Lin Zhang, Yanhong Hou, Mengwei Wang, Benyan Wu, Nan Li

**Affiliations:** 1 Department of Gastroenterology, Second Affiliated Hospital of General Hospital of PLA; Beijing; PR China 100091; 2 Department of Geriatric Gastroenterology, General Hospital of PLA, Beijing 100853, PR China

## Abstract

**Background:**

FBG2 (F-BOX6) gene is an important member in ubiquitin metabolic system F-BOX family, and forms E3 complex with the other members in the family. But its role in gastric cancer is still not clear. In the present study, we intended to investigate the influence of FBG2 on the growth, proliferation, apoptosis, invasion and cell cycle of the gastric cancer line MKN45 and gastric cell line HFE145.

**Methods:**

As a critical component of ubiquitin-protein ligase complex, FBG2 cDNA was subcloned into a constitutive vector PCDNA3.1 followed by transfection in MKN45 and HFE145 by using liposome. Then stable transfectants were selected and appraised. The apoptosis and cell cycles of these clones were analyzed by using flow cytometry. The growth and proliferation were analyzed by cell growth curves and colony-forming assay respectively. The invasion of these clones was tested by using cancer cell migration assay. The FBG2 stable expression clones(MKN-FBG2 and HFE-FBG2) and their control groups were detected and compared respectively.

**Results:**

MKN-FBG2 grew faster than MKN45 and MKN-PC(MKN45 transfected with PCDNA3.1 vector). HFE-FBG2 grew faster than HFE145 and HFE-PC(HFE145 transfected with PCDNA3.1 vector). The cell counts of MKN-FBG2 in the forth, fifth, sixth and seventh days were significantly more than those of others (P < 0.05). Cell cycle analysis showed that MKN-FBG2 and HFE-FBG2 proliferated faster, proportions of cells in G2-M and S were different significantly with control groups (P < 0.05). Results of colony-forming assay showed that the colony formation rates of MKN-FBG2 and HFE-FBG2 were higher than those of control groups (P < 0.05). The results of cell migration assay were all negative.

**Conclusion:**

FBG2 can promote the growth and proliferation of gastric cancer cells and normal gastric cells. It can help tumor cell maintain malignant phenotype too. But it can have a negative influence on the apoptosis or the ability of invasion of gastric cancer cells.

## Background

F-box proteins, a critical component of the evolutionary conserved ubiquitin-protein ligase complex SCF (Skp1/Cdc53-Cullin1/F-box), recruit substrates for ubiquitination and consequent degradation through their specific protein-protein interaction domains. There are five F-box proteins previously identified, such as NFB42 (FBX2), FBG2 (FBX6), FBG3, FBG4 and FBG5. All five proteins are characterized by an approximately 180-aminoid(aa) conserved C-terminal domain and thus constitute a third subfamily of mammalian F-box proteins. FBG2 (F-BOX6) gene is an important member in ubiquitin metabolic system F-BOX family [1,2], and forms E3 complex with the other members in the family. It has been proved in previous researches that ubiquitin metabolic system is an important pathway for the catabolism of some protein molecules in cells, such as products of many oncogenes and anti-oncogenes [3-5], which enter metabolic system through the identification by the members of F-BOX family in E3 complex. It has been confirmed by small interfering RNA that FBG2 is a novel member of F-box protein family which recognizes N-glycans and plays a role in the endoplasmic reticulum-associated degradation (ERAD)[6]. The changes in the expression of FBG2 gene in cells may affect the expression level of some oncogenes or anti-oncogenes so as to influence some biological characters of cells to some degree. Some cDNA gene chips were used to detect the difference in gene expression between gastric adenocarcinoma and the morphologically normal mucosa tissues near carcinoma in our previous research [7,8]. It was found that the expression level of FBG2 gene in carcinoma tissues was higher than that in normal tissues. However, there has been no report on the functions of this gene in gastric cancer cells previously. In this research, gene transfection method was used to introduce FBG2 gene into gastric adenocarcinoma cell strain MKN45 and normal gastric cell strain HFE145, then the cell strains with stable expression were selected out. The changes in the biological characters of the cell strains were detected in order to perform a preliminary analysis on the functions of this gene in gastric cancer cell.

## Methods

### Materials

Gastric adenocarcinoma cell line MKN45 was provided by Shanghai Institute of Biotechnology and preserved by our department. Gastric cell line HFE145 was preserved by our department[9]. FBG2 monoclonal antibody was purchased from Abcam company (USA), PCDNA3.1 vector was preserved by our department, common cell culture plates were purchased from Orange Company(Belgium). Transwell cell culture plates were purchased from Castar Company(USA). AnexinV-FITC apoptosis detection kit was purchased from Beijing Biosea Biotechnology Co., Ltd. All the primers used in this research were synthesized by Shanghai Boya Biotechnology Co., Ltd.

### Expression of FBG2 gene in MKN45 and HFE145

Expressions of FBG2 gene in gastric adenocarcinoma cell strain MKN45 and normal gastric cell strain HFE145 were detected to determine whether the cell lines could used in the research. RT-PCR and immunocytochemical assay were performed to detect the expression of FBG2 in cells, and the results showed that these cell strain were all FBG2 defective cell strains, which were suitable for gene transfection experiment in the research.

### Construction and identification of PC-FBG2 vector

The cDNA of FBG2 gene was obtained by RT-PCR from total RNA of human gastric adenocarcinoma tissues which was used as the templet for PCR. Inner and external primers for nested PCR were synthesized respectively: S: 5' GGGGTACCCCAGGCCATGGATGCTC 3' 129 A: 5' CGGGATCCAACCGGGGCAGGAGTCG 3' 1104 (external primer) S: 5' GGGGTACCATGGATGCTCCCCACTC 3' 136 A: 5' CGGGATCCATGGACAGCTGTCAGAA 3' 1024 (Inner primer)

With the templet of total RNA from gastric adenocarcinoma tissues, nested PCR was performed to obtain the CDS double strand DNA fragments of FBG2 gene with KpnI and BamHI restriction sites in the two ends after two cycles of reactions. KpnI and BamHI were used to incise this double strand fragments and PCDNA3.1 vector. After these incised products were purified, they were kept at 16°C over night for ligation under the actions of T4 ligase. Then the ligated products were used to transform DH_5α _competent cells, and antibiotic screening was performed. PCR identification was conducted to select positive clones. After amplification culture, positive clones were identified by KpnI and BamHI incision. The confirmed positive clones were sent for sequencing, and eukaryon vectors PC-FBG2 with completely correct sequence of FBG2 gene were obtained.

### Transfection of PC-FBG2 vector in MKN45 and HFE145 cells

DMEM culture medium with 10% fetal calf serum was used to culture the MKN45 and HFE145 cells in 12-well cell culture plates until the cells covered 90%–95% of the area. Serum-free DMEM was used for culture over night. Lipofectamine2000 liposome transfection kit was used. According to the directions for use, liposome and PC-FBG2 vector DNA were mixed and added into each well. PCDNA3.1 empty vector transfection group and blank control group (only liposome was added, and there was no vector DNA) were established. Transfection was completed after 24 hours' incubation.

### Selection of cell strains with stable expression of FBG2

Transfected cells were diluted the into 24-well culture plates according to the proportion of 1:20. Then they were selected in medium containing G418. The concentration of G418 was based on the results of preliminary tests (800 μg/ml for MKN45 and 1000 μg/ml for HFE145, the concentration at which there were no surviving cells at 7 days after the time when cells covered 90% of the area of the wells in 6-well culture plate). The selection process continued for 31 days to allow colony formation. Colonies resistant to G418 were isolated with cloning cylinders and transferred into 24-well dishes. 12 and 7 positive clones were respectively obtained in the PC-FBG2 vector transfection group(MKN-FBG2) and PCDNA3.1 empty vector transfection group(MKN-PC) in MKN45 cell line. Then 9 and 5 positive clones were obtained respectively(HFE-FBG2 and HFE-PC) in the two transfection groups in HFE145 cell line. These selected clones were taken for identification and frozen for future use.

### Analysis of transfectants

RT-PCR and Western blotting analysis were respectively performed to detect the mRNA and protein of FBG2, and immunocytochemical analysis was used to detect the expression of FBG2 protein *in situ*.

### Cell growth curve assay

All of 12 MKN-FBG2 cell clones and 9 HFE-FBG2 which stable expressed FBG2 were used. 12 clones which were transfected by PCDNA3.1 empty vector and untreated cell strains were used as control groups. The cells of each clone were inoculated into 24-well culture plate at the concentration of 5 × 10^4^/ml. After the cells completely adhered to the wall, they were washed once with PBS and then trypsinized in 0.5 ml of Trypsin/EDTA and counted in triplicates at 1 to 7 day using a cell counter (Beckman Coulter, Inc., Fullerton, CA). The mean values of all 12 MKN-FBG2 cell clones and 9 HFE-FBG2 on different time were calculated, and growth curves were plotted. In addition, MKN-PC cell clones, HFE-PC cell clones and untreated cell clones were used as control groups.

### Analysis of cell cycle and apoptosis

FBG2 gene stable expression cell groups(MKN-FBG2, HFE-FBG2), PCDNA3.1 empty vector transfection groups(MKN-PC, HFE-PC) and untreated cell control groups were detected by flow cytometry. When the cells covered 70% of the area of cell culture plates in each group, serum-free culture medium was used for synchronization. After 24 hours' continuous culture, the cells were harvested and fixed by 100% ethanol, then prepared for single cell suspensions. After DNA staining, the cell cycles of the samples were measured on a FACS Calibur cytometer. The analysis software was CellQuest. After synchronization and 24 hours' continuous culture, the cells were harvested and fixed, PI and AnexinV-FITC double staining was performed, and flow cytometry was used to detect the apoptosis of cells. 3 replicate tests on every clone were performed in each group, the average values of three groups were calculated respectively, and comparison between three groups was conducted.

### Colony formation assay

MKN-FBG2, HFE-FBG2, MKN-PC, HFE-PC and untreated cell control groups were detected. 1000 cells of each clone were respectively seeded in a 9 cm cell culture dish. After 18 days' culture in DMEM containing fetal calf serum, the number of cell clones with more than 50 cells was counted under microscope in each dash (clone formation rate = number of clones in each dish/1000). Three reduplicate dishes were used from each clone. Cell colonies were then fixed and stained with 0.5% methylene blue (Sigma, Poole, Dorset, U.K.) in ethanol. All colonies visible by eye were counted separately for each sample and evaluated their clone formation rates.

### Cell migration assay

Cell migration assays were performed using FCS-coated polycarbonate filters (8 μm pore size; Transwell)[10]. The membrane undersurface was coated with 200 μl FCS for 1 hr at 37°C and blocked with 200 μl migration buffer (0.5% BSA in DMEM) for 30 min at 37°C. The lower chamber was filled with 500 μl of migration buffer, following which cells were plated in the upper chamber of 4 wells per treatment at a density of 1 × 10^5 ^in 100 μl of migration buffer and incubated at 37°C for 4 hr. Following incubation, cells in the upper compartment were trypsinized and counted by the CASY 1 counter (Sharfe System, Reutingen, Germany). Cells that had migrated to the lower surface of the filter were also trypsinized and counted. The migration rate was obtained by dividing the cell number in the lower chamber by the sum of the cell number found in both the lower chamber and the upper chamber ×100.

### Statistics

SPSS11.0 statistical software was used. Two-factor and one-factor analysis of variance was used for statistical analysis.

## Results

### Expression of FBG2 gene in MKN45 and HFE145 cell lines

**Figure 1:**
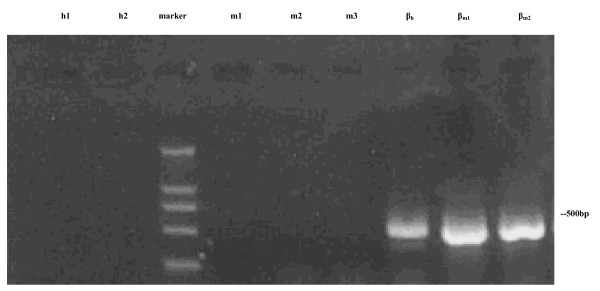
**The results of RT-PCR for FBG2 in MKN45 cell and HFE145 cell**. Note: m1, m2 and m3 were the results of RT-PCR for FBG2 in MKN45 cells, h1, h2 were the results of RT-PCR for FBG2 in HFE145 cells. β_h _was the β-actin control of HFE145 cell, β_m1 _and β_m2 _were β-actin control of MKN45 cells. The results showed that there was not expression of FBG2 gene in MKN45 cell or HFE145 cell.

**Figure 2:**
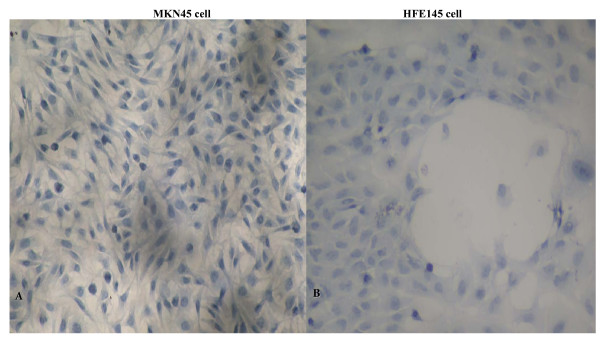
**The Immunohistochemistry results of FBG2 in MKN45 cell and HFE145 cell**. A: There was no postive signal in MKN45 cell. The result showed that there was no expression of FBG2 gene in MKN45 cell. B: There was no postive signal in HFE145 cell. The result showed that there was no expression of FBG2 gene in HFE145 cell too. (×200)

The expressions of FBG2 gene in gastric adenocarcinoma cell strain MKN45 and gastric cell strain HFE145 were detected by RT-PCR and immunocytochemical analysis. All the results in two cell strains were negative, which indicated that there was no detectable expression of FBG2 gene in untreated MKN45 or HFE145 cells. (Figures 1, 2).

### Expression of FBG2 gene in transfectants

**Figure 3:**
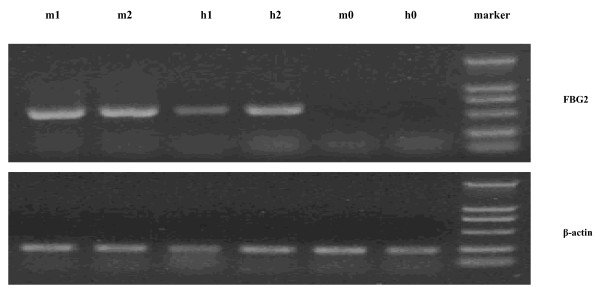
**The RT-PCR results of FBG2 in MKN-FBG2 cell and HFE-FBG2 cell**. Note: m1 and m2 were the results of RT-PCR for FBG2 in MKN-FBG2 cells, h1 and h2 were the results of RT-PCR for FBG2 in HFE-FBG2 cells. M0 was the result of RT-PCR for FBG2 in MKN-PC and h0 was the result of RT-PCR for FBG2 in HFE-PC cells. The results showed that there were expressions of FBG2 gene in MKN-FBG2 cell line and HFE-FBG2 cell line.

**Figure 4:**
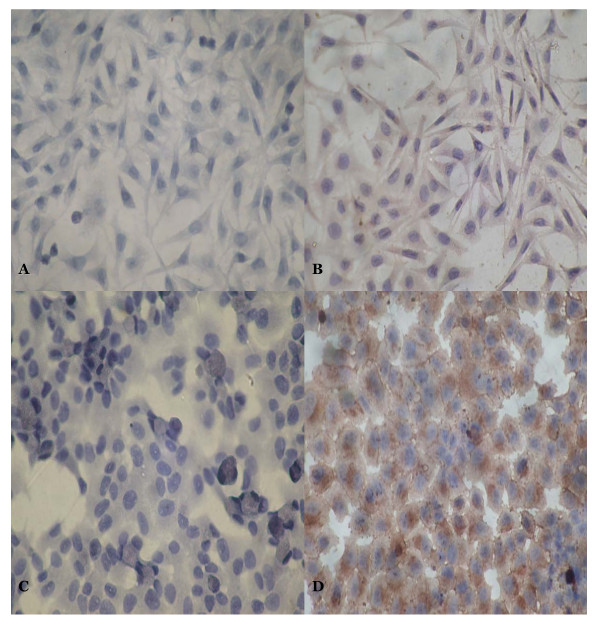
**The immunohistochemistry results of FBG2 in MKN-PC, MKN-FBG2, HFE-PC and HFE-FBG2 cell lines**. A: There was no positive signal in MKN-PC cell. B: There was positive signal in MKN-FBG2 cell. The brown positive signals were mainly distributed in cytoplasm. C: There was no brown positive signal in HFE-PC cell too. D: There was positive signal in HFE-FBG2 cell and the brown positive signals were mainly distributed in cytoplasm and cell membrane. The results showed that there were expressions of FBG2 gene in MKN-FBG2 and HFE-FBG2 cell lines. (×200)

**Figure 5:**
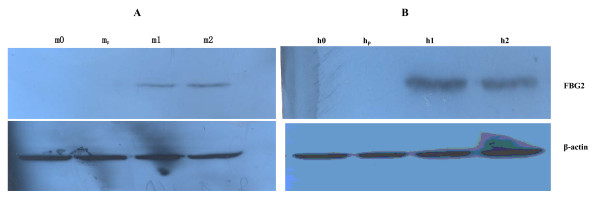
**The results of Western blot for FBG2 in MKN-FBG2, MKN-PC, HFE-PC and HFE-FBG2 cell lines**. A: m1, m2 were the results of Western blot for FBG2 and β-actin in MKN-FBG2 cells with stable transfection of FBG2 and m_p _were those in MKN-PC cells, and m0 was those in MKN45 cells. B: h1, h2 were the results of Western blot for FBG2 and β-actin in HFE-FBG2 cells and h_p _were those in HFE-PC cells, and h0 was those in HFE145 cells. The results showed that there were expressions of FBG2 gene in MKN-FBG2 line and HFE-FBG2 cell line, but no expression in other cell lines.

The expression of FBG2 gene in MKN-FBG2 and HFE-FBG2 cells were detected by using RT-PCR, Western blotting and immunocytochemical analysis. The results of RT-PCR, western blotting and immunocytochemical analysis showed that the expression of FBG2 gene significantly increased in MKN-FBG2 and HFE-FBG2 cells when compared with the untreated MKN45 and HFE145 cells or MKN-PC and HFE-PC cells respectively. On the other hand, the results of immunocytochemical test showed that the expression of FBG2 gene in MKN-FBG2 cells was mainly distributed in cytoplasm and there was no obvious positive signal in cell nucleus and membrane. But the positive signals were mainly distributed in cytoplasm and cell membrane, and there was no obvious positive signal in cell nucleus in HFE-FBG2 cells (Figures 3, 4, 5).

### The influence of FBG2 gene on the growth of cells

**Figure 6:**
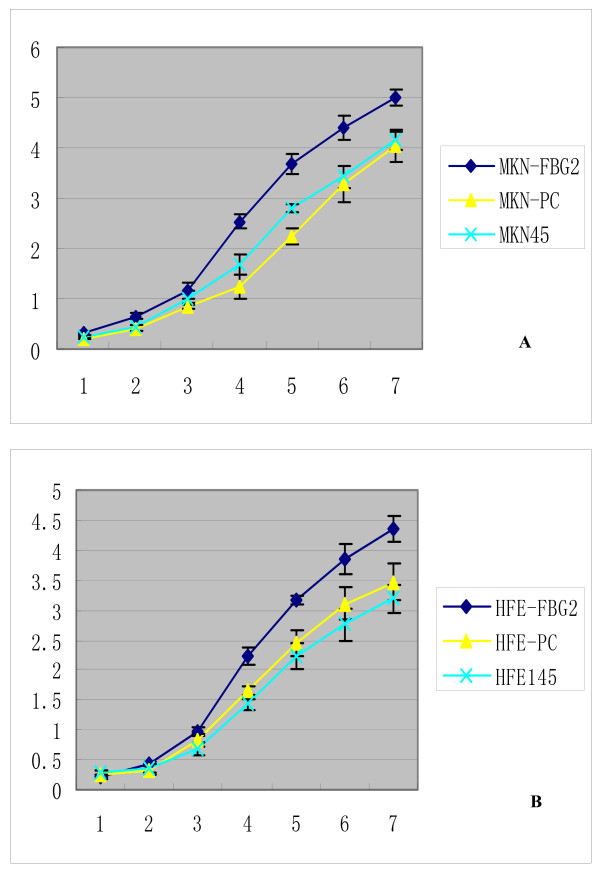
**The growth curves of MKN-FBG2, MKN-PC, MKN45, HFE-FBG2, HFE-PC and HFE145 cell lines**. A: The growth curves of MKN-FBG2, MKN-PC and MKN45 cell lines. The unit of vertical axis was × 10^5 ^that of horizontal axis was the number of days. The results showed that MKN-FBG2 cells grew faster than its control groups. B: The growth curves of HFE-FBG2, HEF-PC and HFE145 cell lines. The results showed that HFE-FBG2 cells grew faster than its control groups too.

The results of cell growth curve assay showed that MKN-FBG2 and HFE-FBG2 cells grew significantly faster than untreated MKN45 and HFE145 cells or MKN-PC and HFE-PC cells respectively (P < 0.05), and there was no significant difference between the control groups (Figure 6). At 4, 5, 6 and 7 days after inoculation, the average cell counts of MKN-FBG2 group were 2.49 × 10^5^, 3.72 × 10^5^, 4.36 × 10^5 ^and 5.01 × 10^5 ^respectively, which were significantly more than those of the two control groups (P < 0.05). The average cell counts at the same days of HFE-FBG2 group were 2.33 × 10^5^, 3.21 × 10^5^, 3.82 × 10^5 ^and 4.63 × 10^5 ^respectively, which were significantly more than those of the two control groups too (P < 0.05).

### Analysis of cell cycle by using flow cytometry

The results of flow cytometry analysis showed that the proportions of the cells in G2-M phase in the MKN-FBG2 and HFE-FBG2 groups were significantly higher than those of the control groups (P < 0.05), the proportions of MKN-FBG2 and HFE-FBG2 cells in S phase were significantly lower than those of the control groups (P < 0.05), and there was no significant difference in the proportions of cells in other phases between them (Table 1, 2). The results of the effect of FBG2 upregulation on individual experiments measuring cell cycle progression were summarized in Tables 1, 2.

**Table 1:** The different cell cycle of MKN-FBG2, MKN-PC and MKN45 group

Group	Clone number	n	G0--G1(%)	G2--M(%)	S(%)
MKN-FBG2	12	3	51.66 ± 7.43	21.71 ± 4.29	26.84 ± 4.18
MKN-PC	7	3	47.84 ± 7.07	5.79 ± 2.3^1^	47.16 ± 6.43^1^
MKN45	1	3	44.58 ± 6.54	3.20 ± 1.58^1^	52.78 ± 6.29^1^

(note: compare with the group of MKN-FBG2, ^1^denoting P < 0.05)

**Table 2:** The different cell cycle of HFE-FBG2, HFE-PC and HFE145 group

Group	Clone number	n	G0--G1(%)	G2--M(%)	S(%)
HFE-FBG2	9	3	66.27 ± 6.96	18.53 ± 6.61	15.22 ± 3.23
HFE-PC	5	3	62.45 ± 8.33	4.04 ± 1.87^(1)^	32.95 ± 8.77^(1)^
HFE145	1	3	71.92 ± 11.18	3.18 ± 0.98^(1)^	27.31 ± 7.02^(1)^

(note: compare with the group of HFE-FBG2, ^(1)^denoting P < 0.05)

### Detection of apoptosis using flow cytometry

The apoptosis assay result showed that the average apoptosis rates of all cell clones in MKN-FBG2 and HFE-FBG2 groups, MKN-PC, HFE-PC groups and untreated MKN45 and HFE145 groups were 1.66 ± 0.24% and 2.32 ± 0.28%, 1.73 ± 0.33% and 2.71 ± 0.47%, 1.78 ± 0.43% and 2.55 ± 0.25% respectively, and there was no statistical significant difference between them (P > 0.05).

### Detection of cell proliferation by using colony formation assay

**Figure 7:**
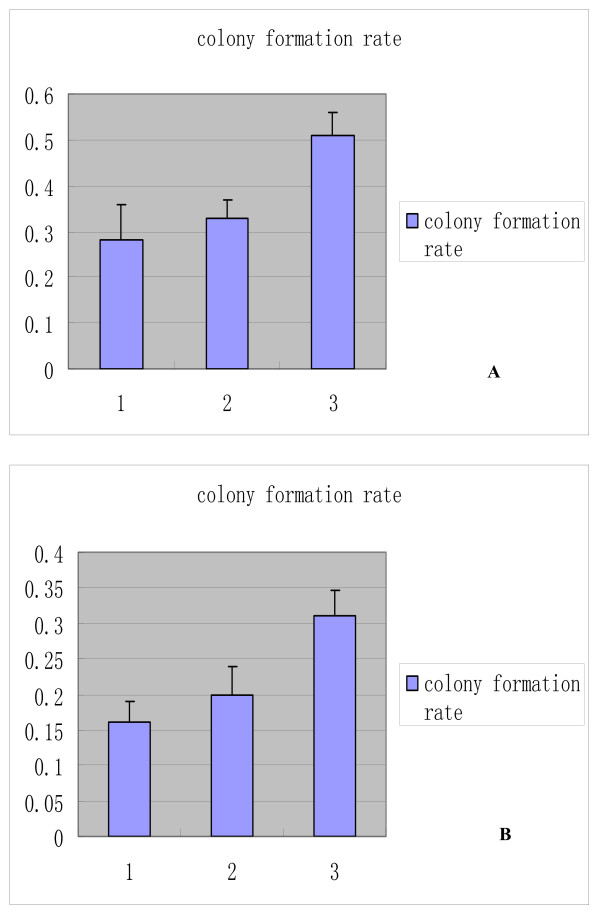
**The result of colony formation assay of MKN45, MKN-PC, MKN-FBG2, HFE-FBG2, HFE-PC and HFE145 cell lines**. A: 1 was the colony formation rate of MKN45 cell line, 2 was that of MKN-PC cell line, 3 was that of MKN-FBG2 cell line. B: 1 was the colony formation rate of HFE145 cell line, 2 was that of HFE-PC cell line, 3 was that of HFE-FBG2 cell line. The results showed that MKN-FBG2 and HFE-FBG2 cells could have a higher proliferative activity than their control groups.

The clone formation rates of the MKN-FBG2 (0.51 ± 0.04) and HFE145(0.32 ± 0.07) group were significantly higher than those of their control groups respectively (P < 0.05). There was no significant difference between these control groups (P > 0.05) (Figure 7). It is apparent that transfection with FBG2 gene increased the capacity of these cells to establish colonies to a highly significant degree.

### The influence of FBG2 gene on the invasion of cells

**Figure 8:**
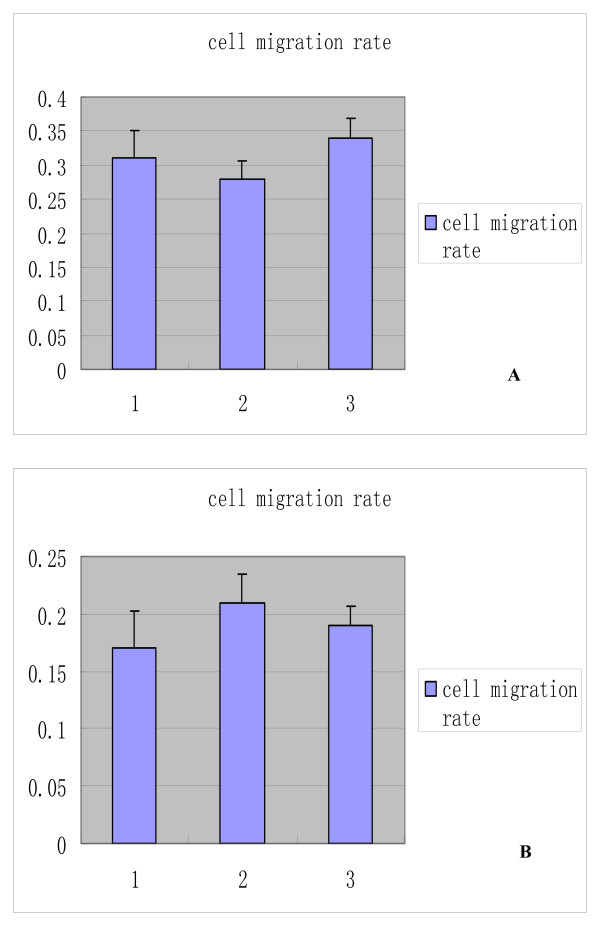
**The result of cell migration assay of MKN45, MKN-PC, MKN-FBG2, HFE-FBG2, HFE-PC and HFE145 cell lines**. A: 1 was the cell migration rate of MKN45 cell line, 2 was that of MKN-PC cell line, 3 was that of MKN-FBG2 cell line. B: 1 was the cell migration rate of HFE145 cell line, 2 was that of HFE-PC cell line, 3 was that of HFE-FBG2 cell line. The results showed that MKN-FBG2 and HFE-FBG2 cells could have not more powerfully invasive activity than their control groups.

Because individual cell migration is an important characteristic of invasive tumor cells, we examined the effects of FBG2 modulation on migration. The results showed that the migration rates of MKN-FBG2, MKN-PC and untreated MKN45 groups were all about 0.3. The rates of HFE-FBG2, HFE-PC and untreated HFE145 groups were about 0.2 (Figure 8). We were unable to observe measurable migration differences in the cell migration experiments. (P > 0.05).

## Discussion

F-box proteins serve as mediators in targeting bound target proteins for ubiquitination and destruction. The ubiquitin-dependent proteolytic pathway plays a key role in the regulation of various short-lived proteins involved in diverse cellular processes in eukaryotes including cell cycle progression, morphogenesis, signal transduction and transcription regulation[11,12]. The primary function of the ubiquitin-dependent proteolytic system is the tagging of substrate proteins with ubiquitin, i.e. covalent attachment of multiple ubiquitin molecules, which allows the proteasome, a 26S protease complex, to recognize and degrade target proteins. This process involves several main steps: (1) activation of ubiquitin in a thioester linkage with ubiquiin-activating enzyme (E1); (2) ransfer of activated ubiquitin from E1 to active site cysteine of one of many ubiquitin-conjugated enzymes (E2s); and finally, (3) conjugation of ubiquitin mainly to acceptor lysine residue of the target protein forming the isopeptide bond[13]. The final step in some cases requires an additional component of the ubiquitin-dependent proteolytic system, ubiquitin-protein ligase (E3), believed to be the most directly involved in target protein recognition and generally composed of several subunits. E3 functions generally as an adapter that interacts with both its cognate E2 and the protein substrate and thus selects this substrate for ubiquitination and consequent degradation. We here describe the roles of one F-box protein named FBG2, which play some roles in many functions of cells with other members in F-BOX family participating in the metabolism of ubiquitin, but there is still lack of research on this gene previously. Some researches [14] showed that F-BOX family participated in the degradation of some anti-oncogenes including P53. The other researches by Wu Qingming, Zhang Weiguo et al [15,16] also showed there was a close relation between the metabolic system of ubiquitin and the proliferation and apoptosis of gastric cancer cells, so it was suspected that the overexpression of the genes of this family might be concerned with the formation and development of gastric cancer. The results of a gene chip research performed by our department also preliminarily confirmed the upregulation of FBG2 in gastric adenocarcinoma tissues. The gene clone technique used in this research further verified its functions in gastric cancer cell line and normal gastric cell line. First, liposome mediated gene transfection and G418 pressure screening were used to obtain cell strains with stable transfection of FBG2 genes, which were verified by immunocytochemistry, RT-PCR and Western blotting analysis. Growth curve assay, colony formation assay and flow cytometry assay were performed for the functional verification on the gastric cancer cell line and gastric cell line with stable expression of FBG2. The results of growth curve assay and colony formation assay showed that the growth and proliferation of the cell strains with stable expression of FBG2 were significantly faster than those of the cells transfected with empty vectors and untreated control cells not only in gastric cancer cell line but also in normal gastric cell line. Therefore, FBG2 gene could accelerate the growth and proliferation of cells. The reasons might be as follows: (1) The gene products promoted the activities of the metabolic system of ubiquitin so as to enhance the metabolism of some protein molecules in cells and accelerate the growth and proliferation of cells. (2) It might accelerate the degradation of proteins inhibiting the growth and proliferation of cells so as to promote the growth and proliferation. The results of flow cytometry assay showed that the proportions of cells in G2-M phase in the cell strains with stable expression of FBG2 were higher than those of the control groups and the proportions of cells in S phase were lower than those of the control cells. Corinna Benz[17] thought that F-box proteins could control cell cycle by adjusting the degradation of some proteins which controlled cell cycle such as cyclins. The division cycle of eukaryotic cells is controlled by protein kinases which are activated by binding to cyclins. Cyclins are present only at particular times in the cell cycle; after they are no longer required they are destroyed by ubiquitination followed by digestion by the proteasome [18-20]. The ubiquitin chains are added by a cascade of enzymes called E1, E2 and E3 ubiquitin ligases, and specificity is determined by the E3 components. The E3 ligases that are important for cell cycle control are the anaphase promoting complex or cyclosome (APC/C) and the Skp1-Cdc53/cullin-F-box protein (SCF) complex [21]. In SCF complexes, proteins with an "F-box" domain (also called "cyclin-like F-box") link targets to the degradation machinery.

There was no significant difference of the apoptosis rates between each group. The results indicated that for the cell strains with stable expression of FBG2, many were in the division stage, so FBG2 gene could accelerate the growth and proliferation of cells. However, this gene did not affect the apoptosis of gastric cancer cells or normal gastric cells perhaps because FBG2 gene or the metabolic system of ubiquitin had little influence on the key genes concerned with apoptosis procedure. The results of Transwell migration assay showed that there was no significant difference in the migration capacity, which represented the invasiveness of cells, between each groups of these cells and the cause needed to be further investigated. A probable cause was that there was no close relation between the gene products (proteins) concerned with invasion and FBG2 gene or the metabolic system of ubiquitin. The main conclusion of this research is as follows: FBG2 gene can significantly promote the growth and proliferation of gastric cancer cells and normal gastric cells and change the cell cycle of them. There were still many deficiencies in our research. For example, only a few cell lines were used. In future researches, the cell lines with high expression of FBG2 gene will be used for RNAi or antisense and ribozyme expression inhibition in order to further verify the functions. Our extensive attempts are to find the capital ligands and functional route of FBG2 by proteomics and immunological methods. In addition, animal experiments will also be used to indepthly investigate the relation between FBG2 gene (even the whole F-BOX family and the metabolic system of ubiquitin) and the occurrence and development of gastric cancer.

## Conclusion

The results of the present investigation demonstrated that FBG2 gene is not expressed in MKN45 or HFE145 cell lines. The overexpression of the gene can influence some biological characteristics of gastric cancer cell or normal gastric cell. FBG2 can promote the growth and proliferation of these cells and help tumor cell maintain malignant phenotype. But it can have a negative influence on the apoptosis or the ability of invasion of gastric cancer cells.

## Competing interests

The authors declare that they have no competing interests.

## Authors' contributions

LZ conceived the study, carried out experiments on the transfection and detection and drafted the manuscript. YH carried out experiments on the RT-PCR and Western blot analysis. MW and BW participated in the study design and revised the manuscript. NL used flow cytometry to complete some analysis of cell cycle.
